# Perspectives of Family Caregivers of People With Alzheimer’s Disease on Caregiving Experience and Needs: A Qualitative Study

**DOI:** 10.1192/j.eurpsy.2025.737

**Published:** 2025-08-26

**Authors:** B. Şentürk Kılıç, D. Hiçdurmaz, Y. Ayhan, E. Saka

**Affiliations:** 1 Faculty of Nursing; 2 Faculty of Medicine, Hacettepe University, Ankara, Türkiye

## Abstract

**Introduction:**

Over 55 million people currently have Alzheimer’s disease (AD), with estimates predicting a rise to 78 million by 2030. The burden of AD impacts not only the affected individuals but also their families and society due to the increasing care demands. Caring for people with AD is emotionally and physically taxing. The World Health Organization emphasizes the need for caregiver support.

**Objectives:**

This study aims to understand the caregiving experiences and needs of family caregivers for individuals with AD.

**Methods:**

A qualitative descriptive design was used. Data were collected from 23 caregivers at a university hospital’s geropsychiatric clinic. Participants were family members caring for individuals with AD for at least four hours per day. Interviews were analyzed using content analysis.

**Results:**

**Sample Characteristics:** The mean age of participants was 57.82 and 57% were female. More than 85% lived with the care recipient, the average caregiving duration was 81.34 hours per week. The following themes were extracted from data collected during the interviews (Figure 1).

**Themes:**

**Stuck in Caregiving:** Participants found caring for loved ones with AD to be an unexpected challenge. Many initially missed the onset of AD due to their loved ones’ previous abilities. Family conflicts over caregiving tasks, shaped by differing spiritual and cultural beliefs, were common. Caregivers faced difficulties, especially with stubborn behavior and personal care tasks.

**A Life in Metamorphosis** Most participants experienced profound changes in their personal lives, including social isolation and emotional turmoil. Half of the participants found meaning in caregiving, gaining new perspectives on life.

**Needs:**

Caregivers identified specific needs, including psychosocial support, disease education, in-home care, and adult daycare services.

**Image 1:**

**Figure:**
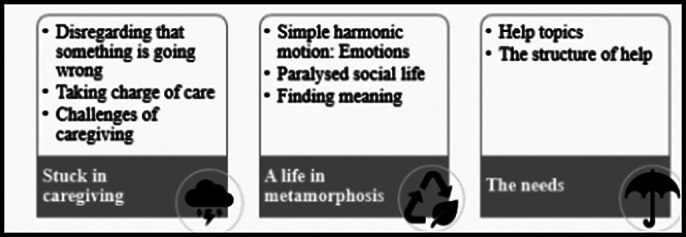

**Conclusions:**

The gradual onset of AD delays symptom recognition, making diagnosis hard for families to accept. As caregiving demands increase, conflicts between caregiving and personal responsibilities cause stress and neglect of personal life. Despite these challenges, many caregivers find personal growth. They seek temporary care options, home assessments, and improved access to education and counseling, highlighting areas for better caregiver support.

**Disclosure of Interest:**

None Declared

